# Aortic Pseudoaneurysm Presenting As Pneumonia and Hemoptysis: An Atypical Presentation

**DOI:** 10.7759/cureus.80618

**Published:** 2025-03-15

**Authors:** Hassan H Quadri, Feras Beitar, Yousof O Rayes, Osman Ahmedfiqi

**Affiliations:** 1 Internal Medicine, Prince Mohammed Bin Abdulaziz Hospital, Ministry of National Guard Health Affairs, Madinah, SAU

**Keywords:** aortic pseudoaneurysm, chest pain, hemoptysis, tb, widened mediastinum

## Abstract

We report a case of an 83-year-old female patient, known to have multiple comorbidities, who presented to our hospital with pleuritic chest pain, productive cough with yellowish sputum and hemoptysis. Chest X-ray demonstrated left upper lobe infiltrate with high inflammatory markers. The patient was admitted as a case of community-acquired pneumonia with suspicion of pulmonary tuberculosis (TB). QuantiFERON-TB and *Mycobacterium tuberculosis* polymerase chain reaction (PCR) with septic workup, including blood and urine cultures, were sent; intravenous antibiotic was started empirically. During the patient’s hospital course, her hemoptysis worsened, and she was ultimately diagnosed with a thoracic aortic pseudoaneurysm with an associated surrounding hematoma.

## Introduction

Aortic pseudoaneurysm can be defined as a confined rupture of the aorta wherein the majority of the wall of the aorta has been breached, and luminal blood is contained solely by the thin rim of the residual wall or adventitia [1]. Thoracic aortic pseudoaneurysms are very uncommon but can be serious and cause a mortality of 32% to 40% in aneurysmal rupture [2]. A systematic review and meta-analysis done by Gouveia e Melo et al. showed an incidence of just 5.3 per 100,000 individuals per year and prevalence of only 0.16%, and the incidence of ruptured thoracic aortic aneurysms was 1.6 per 100,000 persons [3].

Thoracic aortic pseudoaneurysm can be caused by blunt trauma to the chest, cardiothoracic surgery and connective tissue disorders [4]. Patients with thoracic aortic pseudoaneurysm usually don't experience any symptoms unless the aneurysm enlarges enough. Therefore, they are found incidentally on routine chest radiographs [5]. Depending on the magnitude and site of the aneurysm, symptoms, if present, may differ from dysphagia, hemoptysis, dyspnea, and hoarseness to recurrent pneumonitis [6]. Typical presentation is pain in the chest and back region. Others may complain of cough, hoarseness of voice or shortness of breath. Moreover, some can present with signs and symptoms of stroke and loss of consciousness [7]. Few can present with dysphagia if the aneurysm exerts pressure on the esophagus [8]. Hemoptysis as a clinical manifestation is very rare in aortic aneurysms [9].

As patients can be asymptomatic or present with a vague array of symptoms, it is important to consider thoracic aortic pseudoaneurysm as one of the differentials, as early detection of pseudoaneurysm and prompt treatment can be life-saving [1].

## Case presentation

An 83-year-old female patient presented with a known case of type 2 diabetes mellitus on insulin and dapagliflozin 10 mg. She is also hypertensive, taking bisoprolol 2.5 mg and amlodipine 5 mg. Moreover, she has a history of ischemic heart disease (IHD) status post percutaneous coronary intervention (PCI) six years ago on atorvastatin 40 mg, atrial fibrillation on apixaban 2.5 mg, chronic kidney disease (CKD) stage 4 on furosemide 20 mg and acute on chronic calcular cholecystitis status post laparoscopic cholecystectomy.

She presented with complaints of pleuritic chest pain radiating to both shoulders and back, accompanied by diaphoresis for one week. One day, she developed hemoptysis. She had a history of productive cough (yellow sputum) for two months mixed with blood. Additionally, there were complaints of abdominal pain and constipation, with repeated episodes of vomiting food content, reduced oral intake and dysphagia to both solids and liquids. On examination, the patient looked ill and mildly dehydrated, conscious, alert and oriented. Glasgow Coma Scale (GCS) was 15/15; she was vitally stable with blood pressure (BP) 152/77 mm Hg and maintaining 92% oxygen saturation (SpO2) on one litre nasal cannula. Chest auscultation demonstrated equal bilateral air entry with bilateral basal crepitations; cardiovascular examination was unremarkable; abdomen was soft and lax, distended with generalized tenderness but no bruit. There was mild bilateral pitting edema in lower limbs with no signs suggestive of deep vein thrombosis (DVT), tenderness or rash, with equal diameter of both legs. The electrocardiogram (ECG) showed normal sinus rhythm with no ischemic changes or arrhythmias.

**Figure 1:**
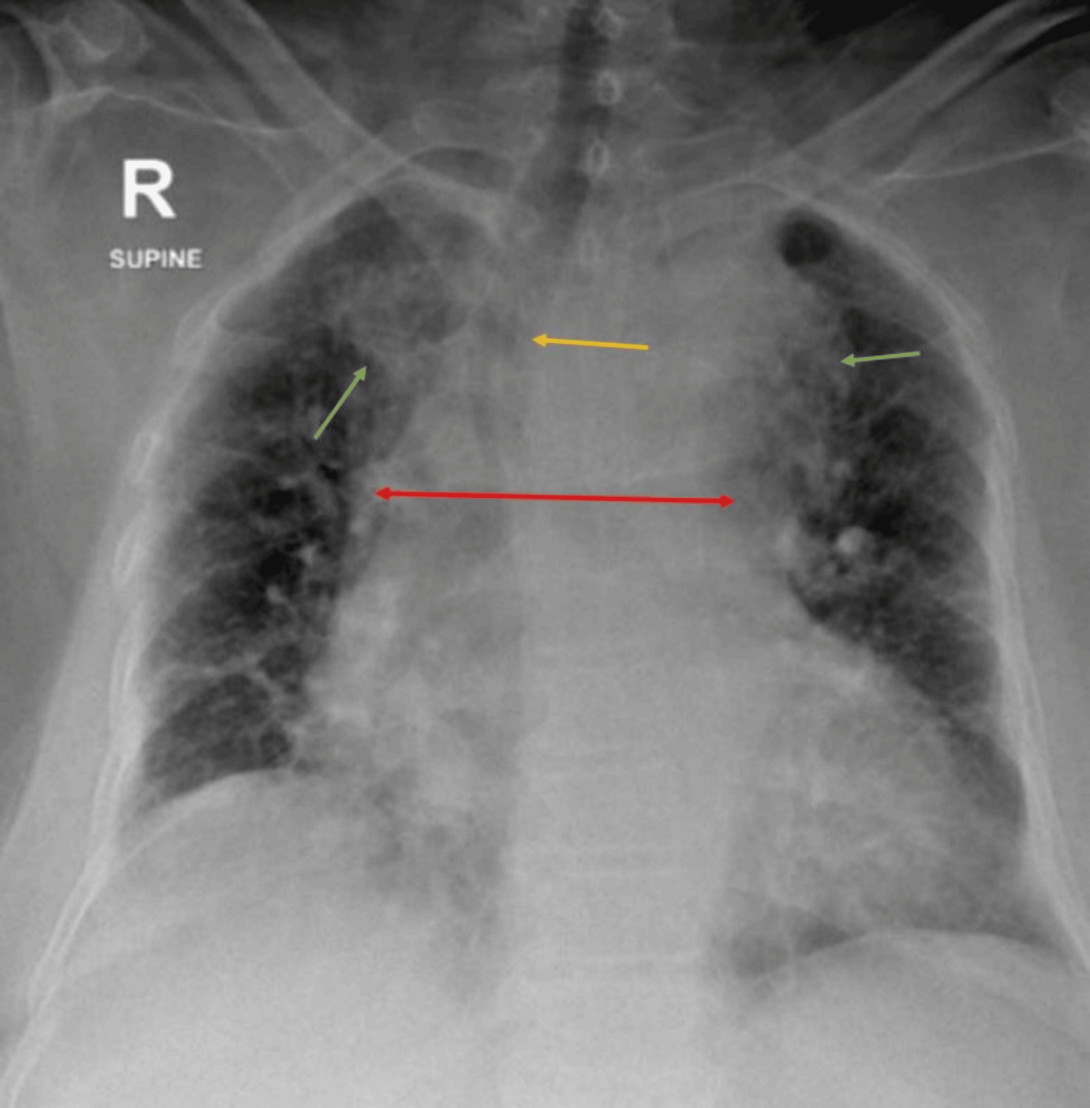
Chest X-ray taken on day 1 post-admission, showing bilateral upper lobe consolidation (green arrows), mediastinal widening (red arrow), and a trachea shifted to the right side (yellow arrow)

Laboratory investigations performed after admission in the hospital showed normocytic anemia mostly attributed to CKD, high inflammatory markers suggesting pneumonia/tuberculosis (TB), acute kidney injury (AKI) on top of pre-existing CKD, elevated D-dimer mostly due to her infection, and mildly elevated brain natriuretic peptide (BNP) (Table 1). Venous blood gas (VBG) showed mixed acid base disturbance of metabolic acidosis with respiratory acidosis (Table 2). Urine analysis was suggestive of urinary tract infection (UTI) (Table 3), with positive urine culture for *Candida albicans* >100.000 CFU/mL. Chest X-ray revealed upper lobe consolidation bilaterally with mediastinal widening and trachea shifted to the right side (Figure 1).

**Table 1:** Relevant laboratory investigations upon admission Hgb: hemoglobin; MCV: mean corpuscular volume; RBC: red blood cells; WBC: white blood cells; CRP: C-reactive protein; INR: international normalized ratio; PT: prothrombin time; PTT: partial thromboplastin time; GFR: glomerular filtration rate; BNP: brain natriuretic peptide

Investigation	Patient’s value	Normal value
Hgb (gm/L)	117	120-160
MCV (fL)	95.6	76-96
RBC (x10^9^/L)	3.88	4-5.4
Platelet (x10^9^/L)	586	150-450
WBC (x10^9^/L)	14.7	4-11
Neutrophils (x10^9^/L)	11.8	2-7.5
CRP (mg/L)	244.8	0.1-4.9
INR	1.14	0.8-1.2
PT (seconds)	13.2	13.5
PTT (seconds)	27.6	26-41
GFR (mL/min/1.73m^2^)	12	90-120
Creatinine (µmol/L)	329	50-98
Troponin I (µg/L)	0.001	<0.015
D-dimer (mg/L FEU)	4.07	0-0.5
Lactic acid (mmol/L)	0.8	0.51-2.1
BNP (pg/mL)	133	<99

**Table 2:** Venous blood gas (VBG) analysis: metabolic and respiratory acidosis

Test	Patient’s value	Normal value
pH	7.17	7.31-7.41
pCO_2_ (mmHg)	52	41-51
HCO_3_^- ^(mmol/L)	16.6	23-29

**Table 3:** Urine analysis The "+" symbols indicate concentration levels, with higher numbers representing a greater presence of the substance in urine.

Test	Result
Glucose	++++
Ketone	Negative
Blood	Trace
Leukocyte esterase	250
Nitrite	Negative
Protein	+
RBC	>30/high power field (HPF)
WBC	>30/HPF
Squamous epithelial	0-2/HPF
Yeast	4+

The patient was admitted for further investigations as a case of community-acquired pneumonia with suspected TB, UTI and AKI on top of CKD. She was empirically started on intravenous piperacillin/tazobactam and later on escalated to meropenem for better coverage as previous urine culture was positive for *Escherichia coli* extended-spectrum β-lactamase (ESBL). QuantiFERON-TB (interferon gamma release assay) and *Mycobacterium tuberculosis* polymerase chain reaction (PCR) were ordered. AKI on top of CKD workup showed intrinsic kidney injury, for which nephrology was consulted. The patient developed metabolic acidosis with respiratory acidosis, mixed acid base disturbance secondary to kidney disease (acute tubular necrosis) and pneumonia/suspected TB. Nephrology started her on sodium bicarbonate 650mg TID. Her latest VBG showed improvement, with a pH of 7.29, pCO_2_ of 45mmHg, and HCO_3_^-^ of 19.6. The patient's condition did not improve, and her clinical status was deteriorating rapidly, with increased hemoptysis production. The ear, nose and throat (ENT) department was consulted, and they performed bedside fiber optic scope and revealed no upper airway bleeding.

**Figure 2:**
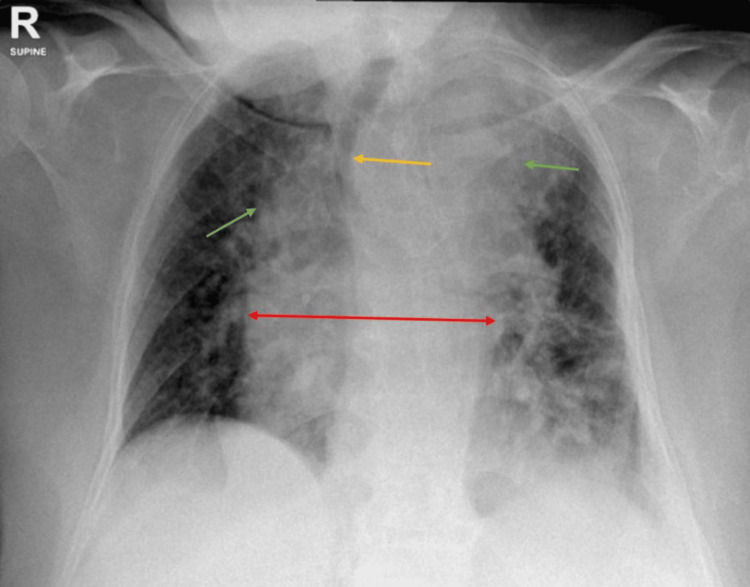
Chest X-ray taken on day 4 post-admission, showing worsening bilateral upper lobe consolidation (green arrows) with mediastinal widening (red arrow) and trachea significantly shifted to the right side (yellow arrow)

A repeat chest X-ray showed a progressive lung infiltrate, mainly in the upper lobes, along with worsening widening of the mediastinum and a significant rightward shift of the trachea (Figure 2).

**Figure 3:**
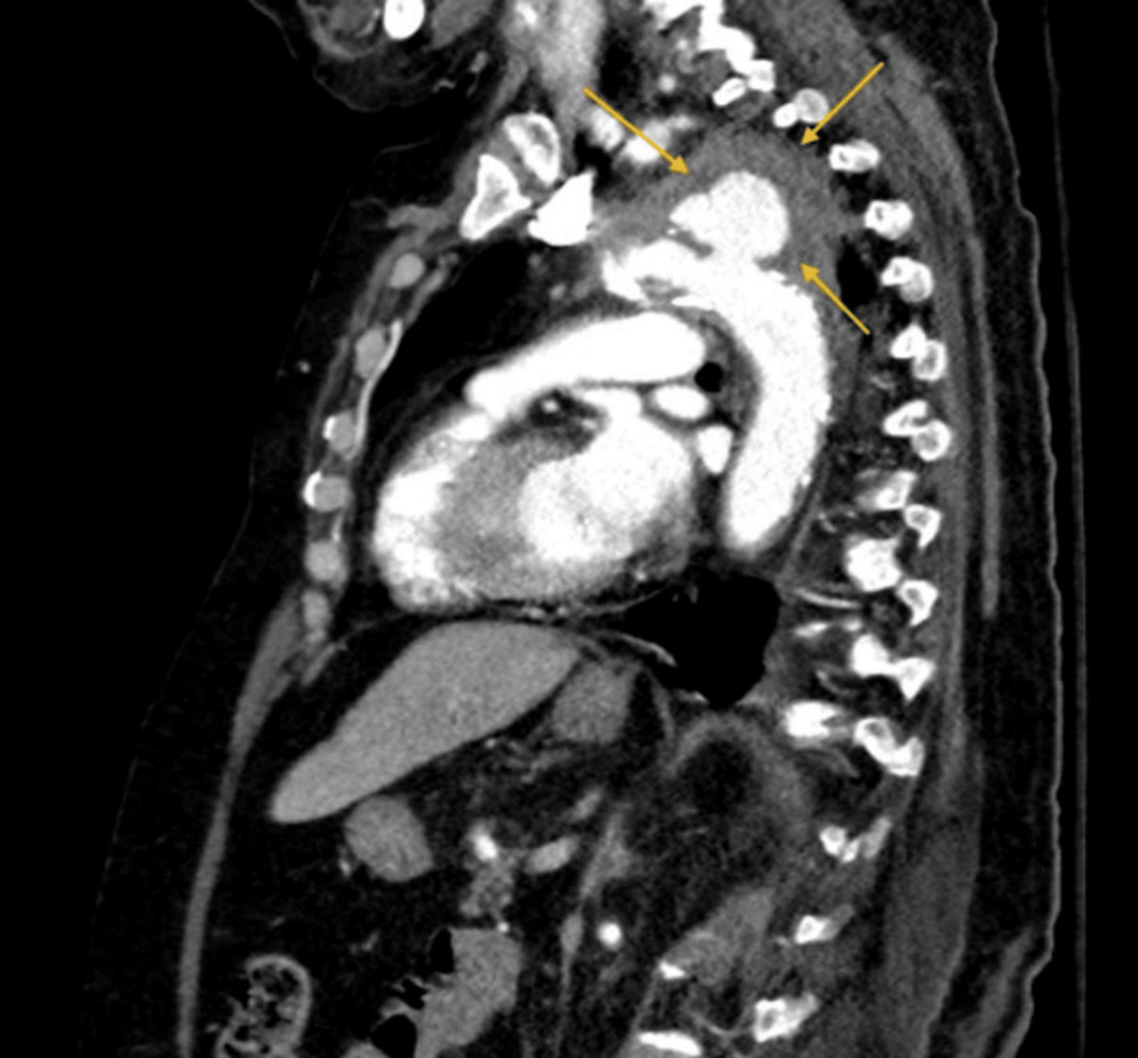
Sagittal view of contrast-enhanced chest CT showing a proximal descending thoracic aorta pseudoaneurysm (yellow arrows) with surrounding hematoma

A suspicion of pulmonary hemorrhage was made, and an urgent chest CT with contrast was ordered. Chest CT demonstrated large irregular focal outpouching arising from the proximal descending aorta (after the origin of the subclavian artery). The sac measured approximately 3.6 x 4.5 x 3.1 cm in anteroposterior (AP), transverse (TR), and craniocaudal (CC) diameters. The neck measured 1.3 cm. It was surrounded by a hematoma. Findings represented a pseudoaneurysm of the proximal descending thoracic aorta with surrounding hematoma (Figure 3).

Vascular surgery was consulted urgently, and she was transferred for life-saving surgical intervention. She underwent surgery on the same night, and a stent graft was inserted. Post-operatively, she required intensive care unit (ICU) admission due to pneumonia and for observation. Eventually, her condition improved, and she was discharged home safely.

## Discussion

This case is reported to highlight the importance and raise awareness that patients with thoracic aortic aneurysm/pseudoaneurysm can present with atypical clinical manifestations, and early diagnosis and treatment are crucial to prevent major complications such as aneurysm rupture and bleeding.

A pseudoaneurysm is a re-perfused pulsatile hematoma which is encapsulated and communicates with the damaged vessel's lumen. It originates when the arterial wall is disrupted [10]. Also called false aneurysms, these arise at the location of arterial injury due to trauma or infection. In a true aneurysm, there is ballooning of the vessel wall (including all layers), but pseudoaneurysm does not include the vascular wall. Blood leaks from the site of injury or infection and is encapsulated by a wall formed due to clotting cascade products. Examples of pseudoaneurysms are cardiac, visceral, femoral and aortic pseudoaneurysms [11].

In this case report, our patient had numerous risk factors for developing an aneurysm/pseudoaneurysm. The patient is female, obese with a body mass index (BMI) of 34.15 kg/m^2^, and has a known history of type 2 diabetes mellitus, hypertension, and IHD, for which she underwent PCI six years ago. Additionally, she was suspected to have an ongoing infection [7,11].

As stated earlier, patients with thoracic aortic pseudoaneurysm can present with a complex nature of symptoms. This occurs due to the indigenous mass effect of the aneurysm itself. Cough is a sign of trachea or bronchus compression. If the mass exerts pressure on the esophagus, patients may complain of dysphagia. Aneurysm can exert strain on the recurrent laryngeal nerve, which can lead to hoarseness of voice. Chest pain, tearing in nature, radiating to the jaw or back, is an initial sign of aortic dissection or rupture [7,8]. Back pain indicates local neurovascular compression, but these generally occur in traumatic pseudoaneurysm patients [12]. Hemoptysis is a very uncommon sign/symptom in thoracic aortic pseudoaneurysm [10]. This can happen if the pseudoaneurysm ruptures and bleeds into nearby structures such as the lung, trachea, and thoracic mediastinum [13]. Azizi et al. reported an interesting case of a young female patient with pseudoaneurysm of thoracic aorta (PTA), complaining of sudden onset of episodic palpitations. She was labelled as PTA presenting with inappropriate sinus tachycardia (IST) due to vagal nerve compression [8].

It is important to discuss the clinical picture of our patient. Our patient had a variety of symptoms, which led to many differential diagnoses. The patient's main complaint was pleuritic chest pain associated with a history of productive cough (yellowish sputum) and hemoptysis. Chest X-ray showed lung infiltrates, and inflammatory markers were elevated. Our initial impression was community-acquired pneumonia and then changed to suspicion of pulmonary TB (based on clinical symptoms of the patient and TB being endemic in Saudi Arabia). Thus, TB and septic workup was ordered, and the patient was empirically started on intravenous piperacillin/tazobactam.

Villar et al. mentioned a case of a 66-year-old male patient who had a similar background of risk factors, comorbidities and clinical presentation as our patient. The patient was known to have uncontrolled diabetes and hypertension and presented with fever, chest pain, shortness of breath, hemoptysis and dysphagia to solids. Similarly, his chest X-ray showed left upper lobe consolidation with trachea shifted to the right side, eventually, he was diagnosed with aortic arch pseudoaneurysm on chest CT angiogram [14].

Likewise, Izzo et al. reported a case where a 58-year-old male patient presented with shortness of breath associated with dry cough, fever and hemoptysis with high WBC count and the chest X-ray was suggestive of right middle lobe pneumonia. The patient was started on intravenous antibiotics, and astonishingly, a pseudoaneurysm of the ascending aorta was discovered on the CT scan [15].

Ansari et al. described a case about a 64-year-old patient presenting with non-productive cough and hemoptysis who was admitted as a case of suspected TB, later diagnosed with thoracic aortic pseudoaneurysm [1]. Similarly, in another case reported by Ikezawa et al., a patient presented with fever for a prolonged duration of time. He was misdiagnosed with pulmonary TB, and he even received anti-TB medications: isoniazid, streptomycin, rifampicin for six months and ethambutol for two months. However, his fever did not subside, and he started to complain of chest and back pain. Consequently, a large descending thoracic aortic pseudoaneurysm was visualized in the chest CT scan [16].

Zhang et al. reported a case of descending thoracic aortic aneurysm with thrombosis where an elderly patient presented to the hospital with a productive cough, sputum mixed with blood for 30 days and sudden development of hemoptysis with no chest pain [17]. Another report in which clinical presentation was similar to the aforementioned case, documented by Millan-Alanis et al., reported a case of an 86-year-old bedridden female patient complaining of hemoptysis for one month. However, upon admission, she was hemodynamically unstable and required resuscitation with crystalloid solutions and blood products. Chest X-ray showed mediastinal widening, and contrast-enhanced chest CT showed an aneurysm of descending and diaphragmatic aorta with a rupture contained by a hematoma. She was treated conservatively due to her multiple comorbidities [18]. Nair et al. mentioned a case of a 65-year-old farmer who complained of blood in sputum for two months, chest X-ray showing widened mediastinum, and CT scan was suggestive of ascending aorta aneurysm [9].

As our patient’s condition was deteriorating and the hemoptysis quantity increased significantly, we decided to consult the ENT department; they ruled out upper airway bleeding with the help of a bedside fiber optic scope. A repeat chest X-ray revealed further widening of the mediastinum with the trachea severely diverted to the right side, which raised the suspicion of pulmonary hemorrhage. Hence, chest CT with contrast was ordered (weighing the benefit vs. risk ratio of contrast-induced nephropathy in CKD patients), which revealed a pseudoaneurysm of the proximal descending thoracic aorta with surrounding hematoma. Consequently, the patient was urgently referred to vascular surgery for prompt surgical intervention, where they inserted a stent graft for her. Post-operatively, after staying in ICU for pneumonia and observation, she was discharged home. Kang and Kang highlighted the significance of contrast-enhanced imaging and early diagnosis of aortic aneurysm rupture to improve surgical outcomes and prognosis of the patient through their case report. He accounts a case of an elderly female who presented to the emergency department with a complaint of hemoptysis for two weeks. They initially misdiagnosed her with left lower lobe lung cancer based on a non-contrast chest CT performed a week before. Likewise our case, her hemoptysis also worsened considerably, and they immediately performed chest CT with contrast in which descending thoracic aorta pseudoaneurysm with hematoma was seen after which she also underwent stent graft insertion [19].

## Conclusions

This case shows the importance of maintaining a high suspicion for life-threatening vascular conditions, in this case, PTA in a patient with non-resolving pneumonia and worsening hemoptysis, despite appropriate antibiotic treatment. Although rare, early recognition of this disease can result in the early prevention of catastrophic outcomes. Our case highlights the need for early imaging with CT angiography, given the atypical clinical progression and worsening hemoptysis, to detect life-threatening vascular conditions and to prevent fatal complications.
